# Baseline atherogenic coefficient and risk of incident chronic liver disease: a prospective cohort study from the CHARLS

**DOI:** 10.1186/s12876-026-05176-8

**Published:** 2026-08-06

**Authors:** Jianteng Dong, Guolin Guo, Tiancheng Meng, Xiaofan Lai, Hongguo Rong, Jian Li

**Affiliations:** 1 School of Traditional Chinese Medicine, Beijing University of Chinese Medicine, Beijing, 102000 China; 2 Center for Evidence-Based Chinese Medicine, Beijing University of Chinese Medicine, Beijing, 102000 China

**Keywords:** Chronic liver disease, The atherogenic coefficient, Prospective cohort study, Metabolic dysregulation, Risk stratification

## Abstract

**Background:**

Chronic liver disease (CLD) is a major public health burden, particularly among middle-aged and older adults. The atherogenic coefficient [(TC − HDL-C)/HDL-C]is a simple lipid-derived index widely used in metabolic risk assessment, but its longitudinal association with incident CLD remains unclear.

**Objective:**

To investigate the longitudinal association between the atherogenic coefficient [(TC − HDL-C)/HDL-C]and the risk of incident chronic liver disease among middle-aged and older adults using data from the China Health and Retirement Longitudinal Study (CHARLS).

**Methods:**

This prospective cohort study used data from the China Health and Retirement Longitudinal Study (CHARLS). A total of 6,721 participants aged ≥ 45 years without CLD at baseline (2011) were followed through 2020. The atherogenic coefficient was analyzed as both a continuous and categorical variable. Cox proportional hazards models were applied to assess associations with incident CLD, and restricted cubic spline models were used to explore nonlinear dose–response relationships.

**Results:**

Among the 6721 participants, 552 participants (8.2%) developed chronic liver disease. Higher baseline atherogenic coefficient was associated with an increased risk of incident CLD. In fully adjusted Cox models, participants in the high atherogenic coefficient group had a significantly higher risk of CLD compared with those in the low-risk group (hazard ratio [HR] = 1.56, 95%CI-1.17-2.06; *p* = 0.002). Restricted cubic spline analyses suggested an overall positive association between the atherogenic coefficient and incident CLD, with the lowest estimated risk observed around an atherogenic coefficient of approximately 3 and a tendency toward higher risk at higher atherogenic coefficient levels (p for overall < 0.001). Subgroup analyses showed broadly consistent associations across examined subpopulations.

**Conclusions:**

A higher baseline atherogenic coefficient may be associated with an increased risk of incident chronic liver disease among middle-aged and older Chinese adults.

**Supplementary Information:**

The online version contains supplementary material available at https://doi.org/10.1186/s12876-026-05176-8.

## Introduction

Chronic liver disease (CLD) is defined by the duration of liver injury (typically lasting ≥ 6 months) and involved sustaining cellular damage that triggers a wound-healing response in the liver, leading to excessive deposition of extracellular matrix (fibrosis); if unmanaged, fibrosis progresses to cirrhosis, and loss of functional liver tissue. Recent estimates suggest that more than 1.5 billion individuals are affected by chronic liver conditions globally, contributing to over 2 million deaths annually [1, 2]. CLD is also a leading cause of liver-related disability-adjusted life years, resulting in considerable healthcare expenditures and long-term functional impairment [3–5]. Given its typically insidious onset and prolonged asymptomatic phase, CLD often remains undiagnosed until advanced stages, underscoring the need for effective strategies for early risk identification and population-level prevention.

Metabolic dysfunction plays a central role in the initiation and progression of CLD [6–8]. Dyslipidemia, insulin resistance, and chronic low-grade inflammation frequently coexist and jointly contribute to hepatic steatosis, inflammation, and fibrotic remodeling [9–12]. In population-based settings, reliance on single metabolic biomarkers may inadequately capture this complex metabolic milieu [13–15]. Consequently, composite lipid-derived indices based on routine laboratory measurements have been increasingly explored for risk stratification in large cohorts [16]. The atherogenic coefficient [(TC − HDL-C)/HDL-C]reflects the balance between atherogenic and anti-atherogenic lipoproteins and has been widely applied in cardiovascular and metabolic risk assessment [17]. Owing to its simplicity, low cost, and availability in routine health examinations, the atherogenic coefficient represents a potentially practical indicator for identifying individuals with an unfavorable metabolic profile at the population level.

Despite growing interest in lipid-derived indices, evidence regarding the longitudinal association between the atherogenic coefficient and the risk of incident CLD remains limited. Most existing studies have focused on cross-sectional associations or cardiovascular outcomes, and few have evaluated long-term liver disease risk in nationally representative cohorts. Moreover, potential nonlinear dose–response relationships between atherogenic coefficient and CLD risk have not been well characterized. Therefore, using data from the China Health and Retirement Longitudinal Study (CHARLS), a nationwide prospective cohort of Chinese adults aged 45 years and older, we aimed to investigate the association between baseline atherogenic coefficient and the risk of incident CLD. We further explored potential nonlinear relationships and examined the discriminatory performance of the atherogenic coefficient across different follow-up periods. We hypothesized that higher atherogenic coefficient levels would be associated with an increased risk of developing CLD during follow-up.

## Materials and methods

### Data source and study population

This prospective cohort study was based on data from the China Health and Retirement Longitudinal Study (CHARLS), a nationally representative population-based cohort of Chinese adults aged 45 years and older initiated in 2011 (http://charls.pku.edu.cn/). The CHARLS survey adopted a multi-stage, stratified probability sampling design proportional to population size, covering 150 counties or districts across 28 provinces in China. The baseline survey was conducted in 2011, with follow-up assessments carried out biennially in 2013, 2015, 2018, and 2020. The baseline response rate was 80.5%, ensuring good representativeness of the study population [18].

**Fig. 1:**
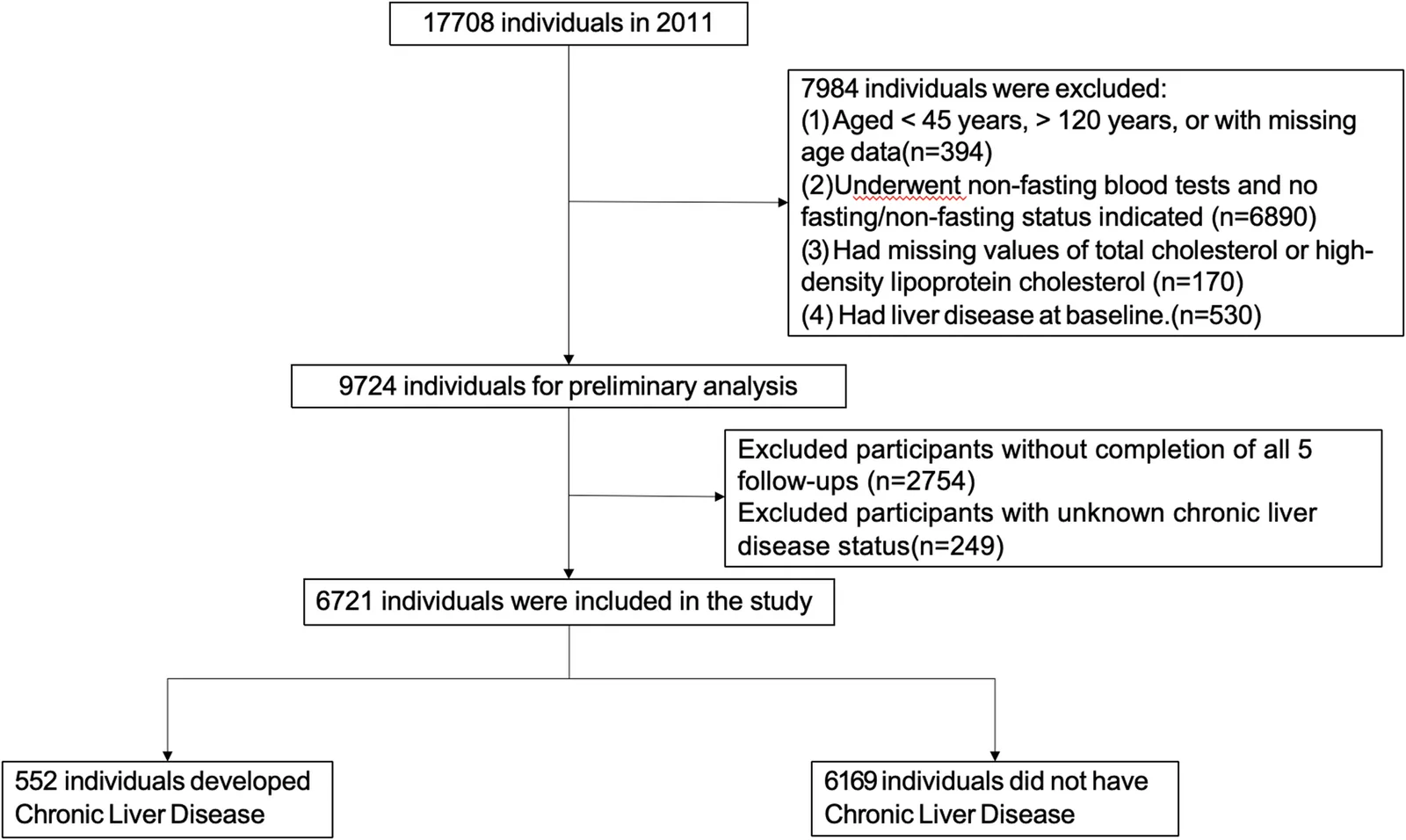
The flowchart of this study

The participant selection process is illustrated in Fig. 1. Of the 17,708 individuals enrolled at baseline in 2011, participants were excluded if they met any of the following criteria: (1) age < 45 years or > 120 years, or missing age information (*n* = 394); (2) non-fasting blood sample collection and no fasting/non-fasting status indicated (*n* = 6,890); (3) missing total cholesterol or high-density lipoprotein cholesterol measurements (*n* = 170); or (4) self-reported CLD at baseline (*n* = 530). After these exclusions, 9,724 individuals were eligible for preliminary analysis. During follow-up, 3,003 participants were further excluded due to incomplete follow-up data. Ultimately, 6,721 participants were included in the final cohort analysis.

The CHARLS study was conducted in accordance with the Declaration of Helsinki and was approved by the Institutional Review Board of Peking University (IRB00001052-11015). Written informed consent was obtained from all participants prior to enrollment. This study adhered to the STROBE guidelines for reporting observational cohort studies.

### The atherogenic coefficient assessment

Fasting venous blood samples were collected at baseline in 2011 and analyzed at the Youanmen Center for Clinical Laboratory of Capital Medical University using standardized enzymatic colorimetric assays. Total cholesterol and high-density lipoprotein cholesterol concentrations were measured from frozen plasma or whole blood samples. The coefficients of variation for lipid measurements were all less than 2%, indicating good analytical reliability. The atherogenic coefficient is calculated as the difference between total cholesterol and high-density lipoprotein cholesterol, then divided by the ratio of high-density lipoprotein cholesterol [19]. The atherogenic coefficient categories were defined with reference to previously published dyslipidemia risk stratification studies and lipid management guidelines evaluating [(TC − HDL-C)/HDL-C]-related cardiometabolic risk. Specifically, participants were grouped as follows: normal group (Atherogenic coefficient < 3.0), mild-risk group (Atherogenic coefficient ≥ 3.0 and < 4.0), moderate-risk group (Atherogenic coefficient ≥ 4.0 and < 5.0), and high-risk group (Atherogenic coefficient ≥ 5.0) [20]. These predefined categories were selected a priori to facilitate comparison with existing literature rather than being derived from the present dataset.

### Assessment of outcome events

The primary outcome of this study was incident CLD. It was defined based on self-reported physician diagnosis obtained from standardized questionnaire interviews. Participants were asked whether a doctor had ever diagnosed them with CLD. Fatty liver disease, liver tumors, and liver cancer were excluded from the outcome definition.

Participants were followed up in 2013, 2015, 2018, and 2020. If a participant reported CLD at any follow-up wave, the event was considered to have occurred at that time point, and the participant was assumed to remain in the CLD state thereafter. Participants without CLD were censored at the last available follow-up. Follow-up time was calculated from the baseline year (2011) to the year of CLD diagnosis or censoring.

### Data collection

The data included in this study were age, sex, body mass index (BMI), smoking status (current smoker or non-smoker), alcohol consumption status (current drinker or non-drinker), household registration type (urban or rural), educational level, and depressive status. Educational level was categorized as illiterate, primary school, secondary education and above, and higher education. Depressive status was assessed using standardized questionnaire items.

All covariates were obtained from baseline interviews conducted in 2011 and were selected a priori based on clinical relevance and previous literature.

### Statistical analysis

Baseline characteristics were summarized using descriptive statistics. Continuous variables were assessed for normality using the Shapiro-Wilk test and are presented as means ± standard deviations or medians with interquartile ranges, as appropriate. Categorical variables are expressed as counts and percentages. Comparisons between groups were conducted using the Mann-Whitney U test for continuous variables and the Chi-square test or Fisher’s exact test for categorical variables. The survey design was specified using the CHARLS sampling weights, primary sampling units, and stratification variables before conducting statistical analyses.

Cox proportional hazards regression models were used to examine the association between baseline atherogenic coefficient and the risk of incident CLD. The atherogenic coefficient was included in the models as both a continuous and a categorical variable. For categorical analyses, atherogenic coefficient was further classified into four risk categories based on predefined cut-off values. Specifically, participants were grouped as follows: normal group (Atherogenic coefficient < 3.0), mild-risk group (Atherogenic coefficient ≥ 3.0 and < 4.0), moderate-risk group (Atherogenic coefficient ≥ 4.0 and < 5.0), and high-risk group (Atherogenic coefficient ≥ 5.0).Three models were constructed: Model 1 was unadjusted; Model 2 was adjusted for age and sex; and Model 3 was further adjusted for body mass index(BMI), smoking status, alcohol consumption, household registration type, educational level, activities of daily living, and depressive status [20].

Restricted cubic spline (RCS) models based on Cox regression were applied to explore potential nonlinear dose–response relationships between the atherogenic coefficient and CLD risk. The number of knots was selected according to the Bayesian Information Criterion, and nonlinearity was assessed using likelihood ratio tests. Adjustment factors in the RCS models were consistent with those used in the Cox regression models.

Kaplan–Meier failure curves were constructed to compare cumulative CLD incidence across the atherogenic coefficient categories during follow-up. Receiver operating characteristic (ROC) curves were generated at 2-, 4-, 7-, and 9-year follow-up intervals to evaluate the predictive performance of baseline atherogenic coefficient for CLD risk.

Subgroup analyses were performed to assess the consistency of the association between the atherogenic coefficient and CLD risk across predefined subgroups, including sex, age group, smoking status, alcohol consumption, household registration type, educational level, and depressive status. Interaction terms were tested to evaluate effect modification.

As a sensitivity analysis, Fine–Gray competing risk regression models were performed with death treated as a competing event. Death information was obtained from follow-up records in the CHARLS database. Corresponding competing risk analyses were conducted for all three Cox regression models, including the unadjusted model (Model 1), the age- and sex-adjusted model (Model 2), and the fully adjusted model (Model 3), to further evaluate the robustness and stability of the observed associations [21].

All statistical analyses were conducted using R software (version 4.3.0) and Stata version 18.0 (Stata Corp). All tests were two-sided, and a p value < 0.05 was considered statistically significant.

## Results

### Characteristics of the study population

Table 1 presents the overall and stratified characteristics of the study participants based on the occurrence of chronic liver disease (CLD) events during the nine-year follow-up period. The overall mean age of the study cohort was 58 years, with females comprising 55.63%. Among the 6,721 participants, a total of 552 individuals (8.2%) developed CLD. The total person-years of follow-up in this cohort study were 58,787, with a mean follow-up duration of 8.75 years. As shown in Table 1, participants in the CLD group exhibited the following characteristics: older age, a higher proportion of males, a higher proportion of smokers and alcohol drinkers, a higher proportion of individuals with rural household registration, and a higher proportion of individuals with impaired activities of daily living (ADL) and depressive symptoms.

**Table 1:** Basic characteristics of the population included in this study

Characteristics	Total Sample	No Chronic Liver Disease	With Chronic Liver Disease	*P *Value
	*N* = 6,721	*n* = 6,169 (91.8%)	*n* = 552 (8.2%)	
Continuous Variables, Mean (SD)				
Age, years	58.307 ± 8.591	58.272 ± 8.643	58.692 ± 7.976	0.271
BMI, kg/m²	23.662 ± 3.957	23.624 ± 3.945	24.110 ± 4.073	0.011
Total Cholesterol, mg/dl	194.796 ± 38.877	194.517 ± 38.302	197.915 ± 44.724	0.049
HDL Cholesterol, mg/dl	51.298 ± 15.225	51.372 ± 15.135	50.475 ± 16.192	0.185
The atherogenic coefficient	3.117 ± 1.659	3.092 ± 1.504	3.401 ± 2.855	< 0.001
Categorical Variables, n (%)				
Risk Level of atherogenic coefficient				0.003
One	3695(54.98%)	3406(55.21%)	289(52.36%)	
Two	1609(23.94%)	1488(24.12%)	121(21.92%)	
Three	826(12.29%)	756(12.25%)	70(12.68%)	
Four	591(8.79%)	519(8.41%)	72(13.04%)	
Age Group, n (%)				0.157
− 45–60 years	4253(63.28%)	3923(63.59%)	330(59.78%)	
- >60 years	2468(36.72%)	2246(36.41%)	222(40.22%)	
Activities of Daily Living (ADL), n (%)				< 0.001
- No Impairment	5680(85.43%)	5251(86.03%)	429(78.72%)	
- Impairment	969(14.57%)	853(13.97%)	116(21.28%)	
Instrumental ADL (IADL), n (%)				0.010
- No Impairment	5465(81.75%)	5037(82.12%)	428(77.68%)	
- Impairment	1220(18.25%)	1097(17.88%)	123(22.32%)	
Gender, n (%)				0.035
- Male	2980(44.37%)	2712(43.98%)	268(48.64%)	
- Female	3737(55.63%)	3454(56.02%)	283(51.36%)	
Education Level, n (%)				0.004
- Illiterate	1937(28.84%)	1815(29.44%)	122(22.18%)	
- Primary School	2720(40.50%)	2481(40.24%)	239(43.45%)	
- Secondary Education	1975(29.41%)	1795(29.11%)	180(32.73%)	
- Tertiary Education	84(1.25%)	75(1.22%)	9(1.64%)	
Marital Status				0.802
Divorced/Separated/Widowed/Never Married, n (%)	937(13.94%)	862(13.97%)	75(13.59%)	
- Currently Married and Living - Together	5784(86.06%)	5307(86.03%)	477(86.41%)	
Current Smoking Status, n (%)				0.226
- Non-smoker	4732(71.50%)	4333(71.30%)	399(73.75%)	
- Smoker	1886(28.50%)	1744(28.70%)	142(26.25%)	
Current Alcohol Use Status, n (%)				0.784
- Non-drinker	4514(67.19%)	4146(67.24%)	368(66.67%)	
- Drinker	2204(32.81%)	2020(32.76%)	184(33.33%)	
Household registration type, n (%)				< 0.001
- Rural	5695(84.75%)	5259(85.26%)	436(78.99%)	
- Urban	1025(15.25%)	909(14.74%)	116(21.01%)	
Depression Status, n (%)				< 0.001
- Non-depressed	4098(63.41%)	3801(64.03%)	297(56.36%)	
- Depressed	2365(36.59%)	2135(35.97%)	230(43.64%)	
Self-Reported Health, n (%)				0.001
Good Health	1477(21.99%)	1388(22.52%)	89(16.12%)	
Poor Health	5239(78.01%)	4776(77.48%)	463(83.88%)	
Life Satisfaction, n (%)				0.001
- Dissatisfaction with Life	908(15.36%)	807(14.88%)	101(20.78%)	
- Subjective Well-being	5003(84.64%)	4618(85.12%)	385(79.22%)	
Chronic disease category, n (%)				< 0.001
No chronic disease	2153(32.03%)	2027(32.86%)	126(22.83%)	
Single chronic disease	2006(29.85%)	1876(30.41%)	130(23.55%)	
Multiple chronic diseases	2562(38.12%)	2266(36.73%)	296(53.62%)	
Number of children category, n (%)				0.108
No children	1710(25.44%)	1579(25.60%)	131(23.73%)	
One child	1639(24.39%)	1512(24.51%)	127(23.01%)	
Two children	1498(22.29%)	1352(21.92%)	146(26.45%)	
Multiple children	1874(27.88%)	1726(27.98%)	148(26.81%)	

Data are presented as means ± standard deviations

*CLD* Chronic Liver Disease; Atherogenic coefficient: the ratio obtained by dividing the difference between total cholesterol and high-density lipoprotein cholesterol by high-density lipoprotein cholesterol. *BMI* Body mass index, *ADL* Activities of daily living

### Association between atherogenic coefficient and CLD risk

After incorporating the atherogenic coefficient as a categorical variable into the Cox regression analyses (Table 2), the normal group (atherogenic coefficient < 3.0) was used as the reference category. In the overall population, participants in the high-risk group showed a significantly increased risk of incident chronic liver disease (CLD) across all models. In the fully adjusted model (Model 3), the hazard ratio (HR) for CLD in the high-risk group was 1.56 (95% CI: 1.17–2.06, *p* = 0.002).

**Table 2:** Cox regression models for the association between the atherogenic coefficient and CLD

Subgroup	Categories	Model 1 (Unadjusted)			Model 2 (Adjusted for age, gender)			Model 3 (Fully adjusted)		
	Risk groups	HR	95% CI	*P*	HR	*95% CI*	*P*	HR	95% CI	*P*
Overall sample	Normal group	1(Reference)			1(Reference)			1(Reference)		
	Mild risk group	0.96	0.78–1.18	0.692	0.97	0.79–1.20	0.787	0.97	0.77–1.21	0.776
	Moderate risk group	1.09	0.84–1.40	0.535	1.10	0.85–1.42	0.486	0.98	0.73–1.30	0.868
	High risk group	1.58	1.23–2.04	0.001	1.60	1.25–2.07	0.001	1.56	1.17–2.06	0.002
		Model 1			Model 2			Model 3’		
Non-drinker	Normal group	1 (Reference)			1 (Reference)			1 (Reference)		
	Mild risk group	1.02	0.80–1.32	0.861	1.03	0.80–1.33	0.809	1.03	0.79–1.35	0.831
	Moderate risk group	1.10	0.81–1.49	0.548	1.10	0.81–1.49	0.559	0.88	0.62–1.27	0.495
	High risk group	1.59	1.16–2.17	0.004	1.61	1.17–2.20	0.003	1.51	1.06–2.14	0.022
Drinker	Normal group	1 (Reference)			1 (Reference)			1 (Reference)		
	Mild risk group	0.83	0.56–1.22	0.346	0.83	0.56–1.21	0.331	0.80	0.51–1.25	0.212
	Moderate risk group	1.08	0.67–1.75	0.757	1.11	0.68–1.79	0.681	1.32	0.80–2.19	0.280
	High risk group	1.58	1.03–2.41	0.037	1.57	1.02–2.42	0.039	1.67	1.03–2.68	0.038

Model 1: unadjusted

Model 2: adjusted for age and sex

Model 3: adjusted for Model 2 plus BMI, smoking status, alcohol consumption, household registration type, educational level, activities of daily living, and depressive status. Model 3’: adjusted for Model 3 without alcohol consumption.

Atherogenic coefficient: the ratio obtained by dividing the difference between total cholesterol and high-density lipoprotein cholesterol by high-density lipoprotein cholesterol.; HR: hazard ratio; CI: confidence interval; BMI: body mass index

Subgroup analyses stratified by alcohol consumption status were further performed. Among non-drinkers, the association between elevated atherogenic coefficient and incident CLD remained generally consistent with the overall analysis. In the fully adjusted model, participants in the high-risk group had a significantly increased risk of CLD compared with the normal group (HR = 1.51, 95% CI: 1.06–2.14, *p* = 0.022).

Among drinkers, a similar trend was observed, although the association was attenuated after full adjustment. In Model 3, the HR for incident CLD in the high-risk group was 1.67 (95% CI: 1.03–2.68, *p* = 0.038). Overall, elevated atherogenic coefficient levels were associated with an increased risk of CLD, and the observed association remained generally stable across different adjustment models and alcohol consumption subgroups.

### Cumulative incidence of CLD

**Fig. 2:**
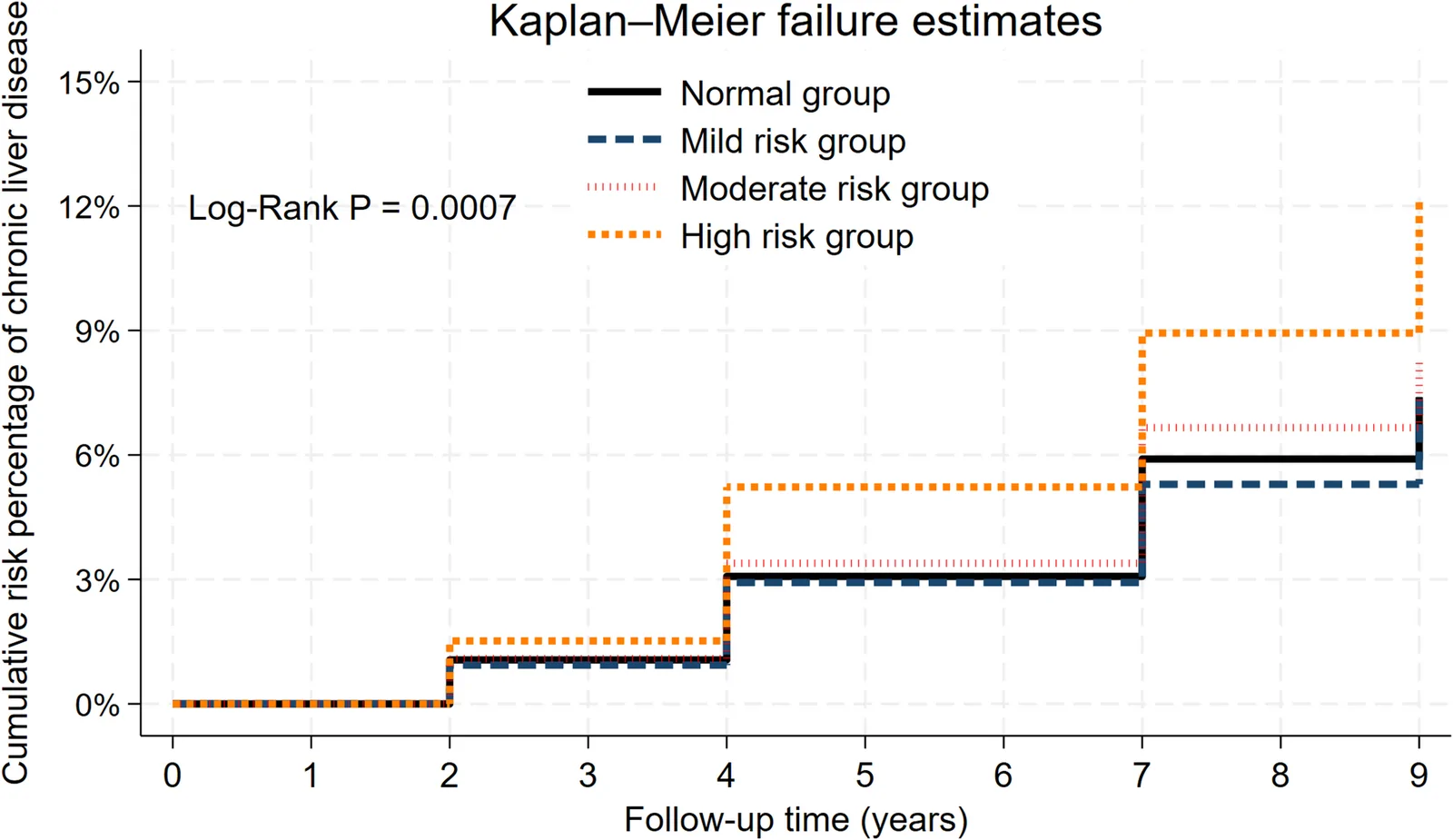
Kaplan–Meier failure curves for cumulative CLD incidence by atherogenic coefficient categories

Kaplan–Meier failure curves were constructed to compare cumulative CLD incidence across atherogenic coefficient categories during follow-up (Fig. 2). Participants with higher atherogenic coefficient categories exhibited a higher cumulative incidence of CLD over time. The separation of curves became more apparent with longer follow-up duration, indicating a sustained difference in CLD risk among atherogenic coefficient groups. (Log-rank test: Chi-square = 14.32, *p* = 0.0007).

### Dose-response relationship assessed by restricted cubic spline analysis

**Fig. 3:**
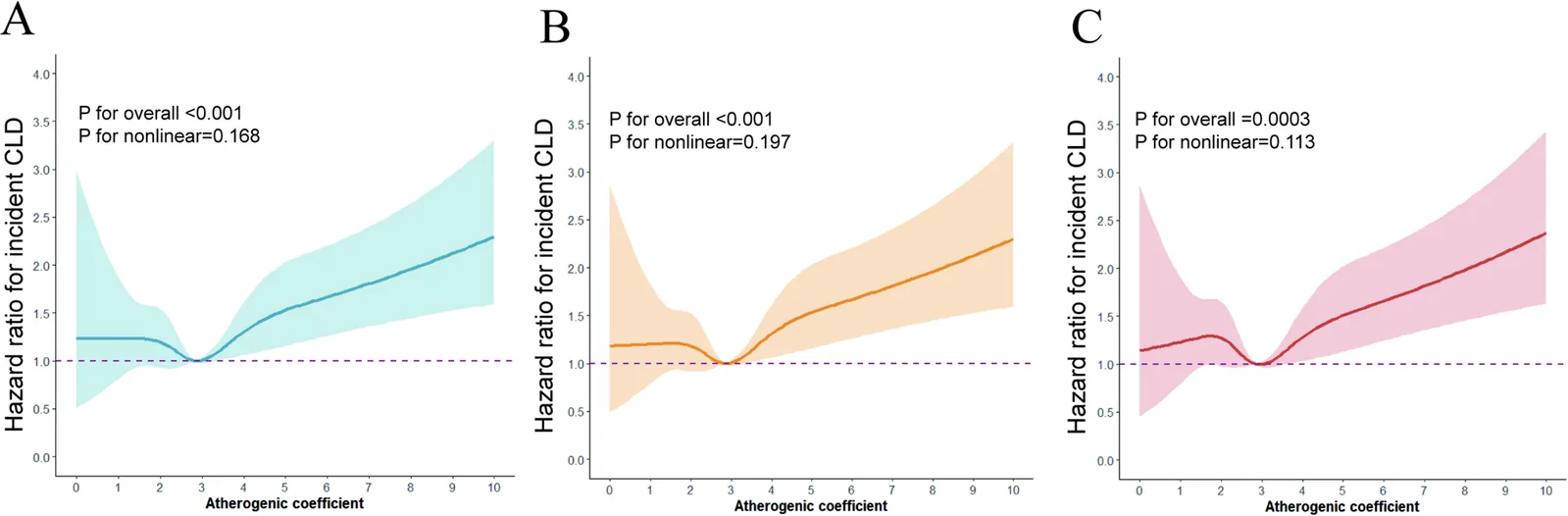
Restricted cubic spline models analyzed the relationship between atherogenic coefficient and CLD. **A** The unadjusted model. **B** The model adjusted for age and sex. **C** The fully adjusted model controlling for age, sex, smoking status, alcohol consumption, household registration type, educational level, activities of daily living, and depressive status

Restricted cubic spline models were used to further explore the dose–response relationship between continuous atherogenic coefficient and CLD risk (Fig. 3). In all models, the overall association between atherogenic coefficient and incident CLD was statistically significant (all p for overall < 0.001). However, the tests for nonlinearity were not statistically significant in the unadjusted model (p for nonlinear = 0.168), the age- and sex-adjusted model (p for nonlinear = 0.197), or the fully adjusted model (p for nonlinear = 0.113).

The spline curves suggested that CLD risk was relatively stable at lower atherogenic coefficient levels and gradually increased at higher levels, with the lowest estimated risk observed around an atherogenic coefficient value of approximately 3. Overall, the restricted cubic spline analyses supported a positive dose–response association between elevated atherogenic coefficient and incident CLD risk, although no statistically significant evidence of nonlinearity was observed.

### ROC analyses of the atherogenic coefficient for incident CLD

**Fig. 4:**
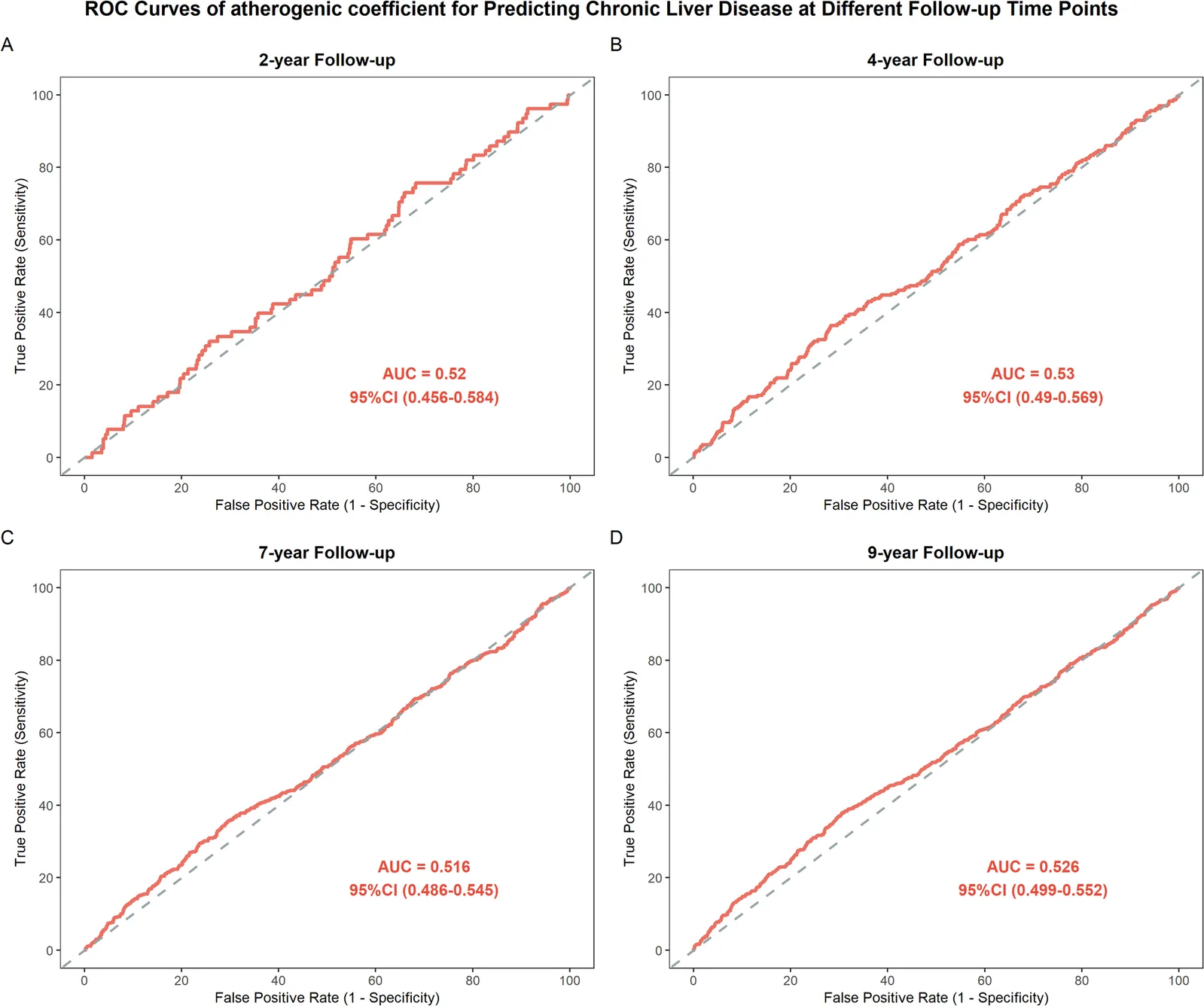
Receiver operating characteristic curves illustrating the discriminatory performance of the atherogenic coefficient for CLD risk at different follow-up time points. **A** ROC curve for 2-year follow-up. **B** ROC curve for 4-year follow-up. **C** ROC curve for 7-year follow-up. **D** ROC curve for 9-year follow-up

Receiver operating characteristic (ROC) analyses were performed to evaluate the discriminatory ability of baseline atherogenic coefficient for incident CLD at 2-, 4-, 7-, and 9-year follow-up intervals (Fig. 4). Across all follow-up periods, the AUC values ranged from 0.516 to 0.530.

Figure 4A shows the ROC analysis of baseline atherogenic coefficient for incident CLD at the 2-year follow-up, with an AUC of 0.520 (95% CI: 0.456–0.584). Figure 4B presents the 4-year follow-up analysis, demonstrating an AUC value of 0.530 (95% CI: 0.490–0.569). During the 7-year follow-up period (Fig. 4C), the AUC value was 0.516 (95% CI: 0.486–0.545). Similarly, the 9-year follow-up analysis (Fig. 4D) yielded an AUC value of 0.526 (95% CI: 0.499–0.552). The ROC analyses suggested limited discriminatory performance of baseline atherogenic coefficient for incident CLD across different follow-up durations.

### Subgroup analysis of the association between atherogenic coefficient and CLD risk

**Fig. 5:**
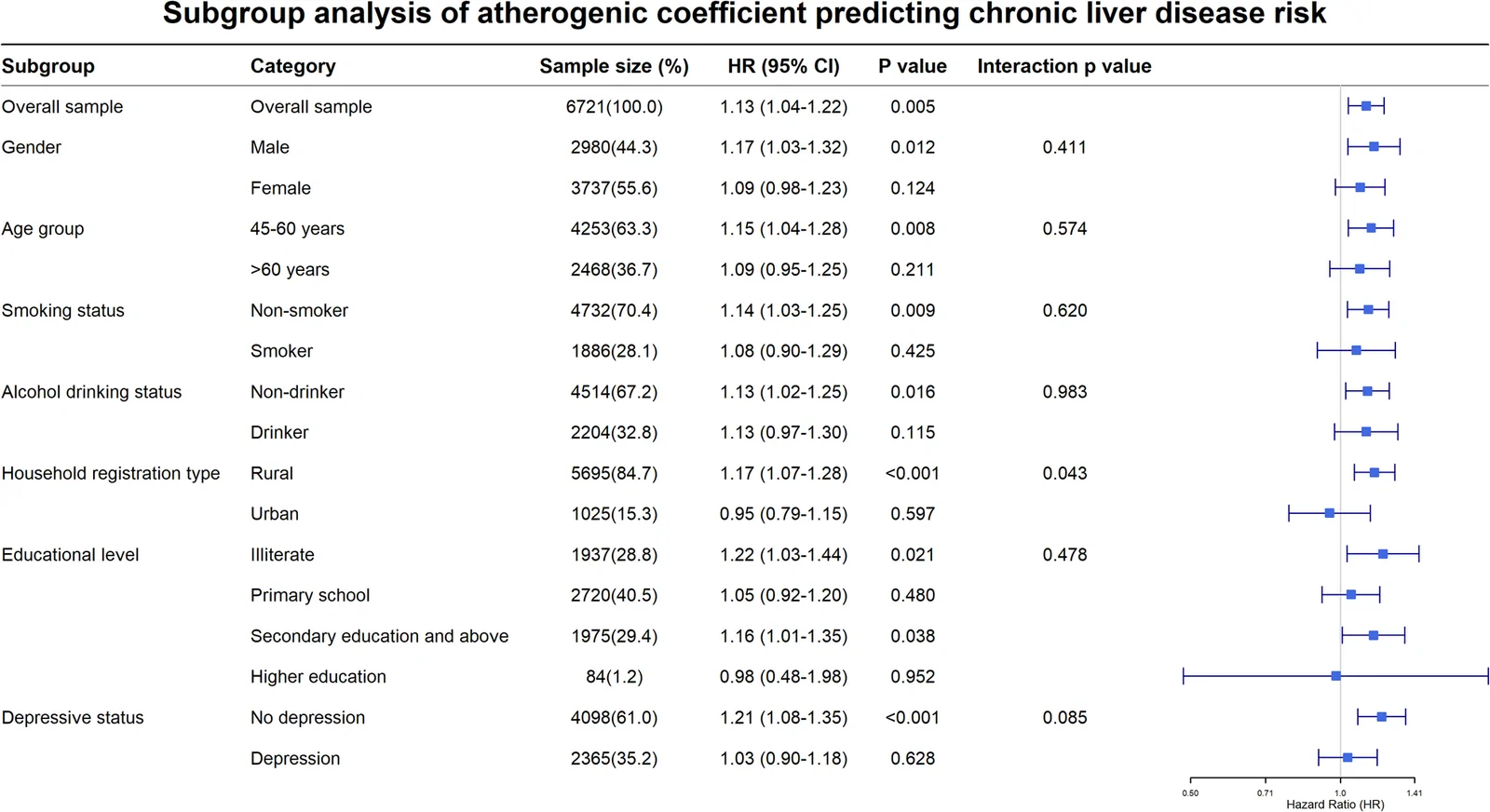
Subgroup analyses of the association between baseline atherogenic coefficient and incident CLD

Subgroup analyses were performed to evaluate the consistency of the association between baseline atherogenic coefficient and CLD risk across different population strata (Fig. 5). In the overall population, a higher atherogenic coefficient was associated with an increased risk of CLD (HR = 1.13, 95% CI 1.04–1.22, *p* = 0.005). The association generally showed a similar direction across subgroups stratified by sex, age group, smoking status, alcohol consumption, household registration type, educational level, and depressive status. Statistically significant associations were observed in several subgroups, including non-smokers (HR = 1.14, 95% CI 1.03–1.25) and rural residents (HR = 1.17, 95% CI 1.07–1.28). Interaction analyses revealed a statistically significant interaction according to household registration type (P for interaction = 0.043), whereas no significant interactions were observed for the remaining subgroup variables (all P for interaction > 0.05). Overall, the association between atherogenic coefficient and CLD risk appeared broadly consistent across most examined subgroups.

### Sensitivity analysis using competing risk regression models

To further evaluate the robustness of the association between atherogenic coefficient and incident CLD, Fine–Gray competing risk regression analyses treating death as a competing event were additionally performed (Table 3). A total of 267 deaths occurred during follow-up and were treated as competing events in the competing-risk analyses. The overall results remained generally consistent with those of the primary Cox proportional hazards models.In the fully adjusted competing risk model (Model 3), participants in the high-risk group exhibited a significantly increased risk of incident CLD compared with the normal group (SHR = 1.52, 95% CI: 1.17–1.97, *p* = 0.002). Similar patterns were observed in the unadjusted and age- and sex-adjusted models.

Overall, the competing risk analyses further supported the robustness and stability of the observed association between elevated atherogenic coefficient and increased CLD risk.

**Table 3:** Fine–Gray competing risk regression analyses for incident CLD treating death as a competing event

Categories	Model 1 (Unadjusted)			Model 2 (Adjusted for age, gender)			Model 3 (Fully adjusted)		
	SHR	95% CI	*P*	SHR	95% CI	*P*	SHR	95% CI	*P*
Risk groups									
Normal group	1 (Reference)			1 (Reference)			1 (Reference)		
Mild risk group	0.96	0.78–1.19	0.730	0.98	0.79–1.20	0.814	0.92	0.74–1.14	0.453
Moderate risk group	1.09	0.84–1.40	0.532	1.10	0.85–1.42	0.486	1.03	0.79–1.35	0.825
High risk group	1.56	1.21–2.01	0.001	1.58	1.23–2.04	< 0.001	1.52	1.17–1.97	0.002

During follow-up, 267 participants died and were treated ascompeting events

Model 1: unadjusted

Model 2: adjusted for age and sex

Model 3: adjusted for Model 2 plus BMI, smoking status, alcohol consumption, household registration type, educational level, activities of daily living, and depressive status

*SHR* Subdistribution Hazard Ratio, *CI* Confidence interval

## Discussion

In this large, nationally representative cohort of middle-aged and older Chinese adults, we found that a higher baseline atherogenic coefficient was associated with an increased long-term risk of incident CLD. This association remained generally stable across multiple adjustment models and was broadly consistent across key population subgroups, including participants without alcohol consumption. Unlike previous cross-sectional studies, the present prospective cohort design enabled evaluation of the temporal relationship between baseline atherogenic coefficient and incident CLD, thereby reducing concerns regarding reverse causality.

Our findings are generally consistent with previous epidemiological studies linking adverse lipid profiles and lipid-derived ratios with liver-related metabolic disorders. Several population-based studies have reported a positive association between atherogenic coefficient and non-alcoholic fatty liver disease (NAFLD), a major component of CLD [22, 23]. For example, a cross-sectional study conducted in a Korean adult population including more than 10,000 participants demonstrated that individuals in the highest atherogenic coefficient quartile had a significantly higher prevalence of NAFLD compared with those in the lowest quartile, with an adjusted odds ratio of approximately 1.6–1.8 [24]. Similarly, analyses from large Chinese health examination cohorts have shown that elevated atherogenic coefficient was associated with increased odds of abnormal liver enzymes, with reported odds ratios ranging from 1.3 to 1.7 after adjustment for demographic and lifestyle factors [25].

In addition, studies examining lipid ratio indices closely related to atherogenic coefficient /HDL-C have provided supportive evidence for the role of dyslipidemia in CLD risk [26, 27]. A large Chinese cohort study involving over 30,000 adults reported that higher triglyceride-to-HDL cholesterol ratios were associated with an increased risk of liver fibrosis markers, with hazard ratios exceeding 1.4 in the highest exposure categories [28]. Although these studies focused on different lipid ratios or intermediate liver-related outcomes, they collectively suggest that lipid imbalance reflected by composite lipid indices is closely linked to chronic liver injury.

Compared with these earlier studies, our investigation extends existing evidence by adopting a prospective cohort design with a mean follow-up of nearly nine years and by evaluating incident CLD rather than cross-sectional liver steatosis or enzyme abnormalities. Importantly, our results showed that the association between atherogenic coefficient and CLD risk remained stable across multiple adjustment models and population subgroups, supporting the robustness of this relationship in a nationally representative sample of middle-aged and older adults. Differences in effect estimates across studies may be attributable to variations in study design, outcome definitions, population characteristics, and follow-up duration rather than fundamental inconsistencies in the underlying association.

Although the observed effect size was modest, the magnitude of association observed for the highest atherogenic coefficient category is comparable to that reported for several established metabolic risk factors for CLD, including obesity and insulin resistance in large Chinese population-based cohorts [29, 30]. In contrast to individual lipid parameters, which often show weak or inconsistent associations with incident CLD after multivariable adjustment, the atherogenic coefficient integrates pro-atherogenic and anti-atherogenic lipoproteins into a single metabolic indicator and demonstrated a stable association across multiple analytical models and alcohol consumption subgroups. The ROC analyses yielded AUC values close to 0.50, indicating limited standalone discriminatory performance, this finding does not negate the statistically significant association observed in the Cox regression analyses. Participants in the highest atherogenic coefficient category remained at an increased risk of incident CLD after multivariable adjustment, suggesting that AC may capture systemic lipid metabolic disturbances relevant to CLD development. Therefore, AC may have epidemiological and metabolic significance as a population-level marker, but it should be interpreted cautiously and should not be used alone for individual clinical decision-making.

Because physician-diagnosed CLD was self-reported, underdiagnosis and outcome misclassification are possible, particularly for individuals with asymptomatic or early-stage disease. Such misclassification is likely to be non-differential with respect to baseline atherogenic coefficient, which would generally bias effect estimates toward the null rather than create spurious positive associations. Therefore, the true association may have been underestimated. Similar outcome definitions based on self-reported physician diagnosis have been widely adopted in large longitudinal population-based cohorts, including CHARLS and other internationally recognized aging studies such as the U.S. Health and Retirement Study (HRS) and the English Longitudinal Study of Ageing (ELSA), where objective imaging and laboratory assessments are not routinely available [31–33]. Nevertheless, future studies incorporating imaging findings, laboratory biomarkers, electronic health records, or validated clinical diagnoses are warranted to further confirm these observations.

Several potential biological mechanisms may underlie the observed association. An elevated atherogenic coefficient reflects an imbalance between atherogenic and protective lipoproteins and is often indicative of broader metabolic dysfunction [34]. Endothelial dysfunction has increasingly been recognized as one of the earliest events during CLD development. Liver sinusoidal endothelial cells (LSECs) undergo capillarization before the activation of hepatic stellate cells and collagen deposition, thereby promoting inflammation, fibrosis, and progressive liver injury. Previous studies have demonstrated that atherogenic lipid abnormalities are closely associated with systemic endothelial dysfunction, impaired nitric oxide bioavailability, oxidative stress, and chronic vascular inflammation. As an integrated lipid-derived indicator reflecting the balance between atherogenic and anti-atherogenic lipoproteins, the atherogenic coefficient may therefore capture early metabolic alterations associated with endothelial injury [35–37]. Elevated cholesterol-related lipotoxicity may promote hepatocellular stress responses, mitochondrial dysfunction, inflammatory cytokine production, and oxidative damage, thereby facilitating persistent hepatic injury and progressive liver dysfunction [38–40]. In addition, low HDL-C levels may impair reverse cholesterol transport and reduce the anti-inflammatory and antioxidant protective effects of HDL particles, further aggravating systemic and hepatic metabolic imbalance [41–43].Moreover, dyslipidemia-related metabolic disturbances are not limited to a single liver disease subtype and may broadly influence susceptibility to multiple chronic hepatic conditions through shared mechanisms involving inflammation, immune dysregulation, endothelial dysfunction, and altered lipid homeostasis [44–47]. Therefore, the atherogenic coefficient may serve as a surrogate marker reflecting overall metabolic and hepatic risk burden at the population level.

From a public health perspective, the atherogenic coefficient has several advantages, including low cost, routine availability, and ease of interpretation. As a simple lipid-derived indicator, it may provide complementary information on systemic lipid metabolic imbalance at the population level. Given its limited standalone discriminatory performance, the atherogenic coefficient should be interpreted cautiously and should not be used alone for individual clinical decision-making.

This study has several notable strengths. First, it is based on the CHARLS cohort, a large, nationally representative population-based study, enhancing the generalizability of our findings among middle-aged and older adults in China. Second, the relatively long follow-up period allows for the assessment of long-term risk associations. Third, the consistency of results across multiple statistical models, including Cox regression, restricted cubic spline analysis, and subgroup analyses, supports the robustness of the observed association.

Several limitations should be acknowledged. First, incident CLD was identified based on self-reported physician diagnosis rather than objective clinical assessments. Because CLD may remain asymptomatic for prolonged periods, outcome misclassification may have occurred and potentially attenuated the observed associations. Second, fatty liver disease, liver tumors, and liver cancer were excluded according to the original CHARLS disease classification framework. As fatty liver disease is closely associated with dyslipidemia and metabolic dysfunction, excluding these conditions may underestimate the overall liver disease burden related to adverse lipid profiles and limit the generalizability of the findings. Third, although multiple covariates were adjusted for, residual confounding related to chronic hepatitis B virus infection, detailed alcohol exposure, liver-related biomarkers, and lipid-lowering medications cannot be completely excluded. Furthermore, because CLD status was assessed at discrete follow-up waves, the exact timing of disease onset was unavailable. Consequently, event times were interval-censored, whereas standard Cox proportional hazards models assume precisely observed event times. This limitation may have influenced the estimation of hazard ratios. Finally, although the atherogenic coefficient was independently associated with incident CLD, its standalone discriminatory performance was limited, indicating that it should not be interpreted as an individual clinical prediction tool.

Despite these limitations, the present findings provide population-level epidemiological evidence supporting a longitudinal association between elevated atherogenic coefficient and increased CLD risk. Further prospective studies incorporating imaging evaluations, laboratory biomarkers, and etiologically classified liver disease outcomes are warranted to better clarify the underlying mechanisms and clinical implications.

## Conclusion

From a public health perspective, our findings suggest that the atherogenic coefficient, a simple and readily available lipid-derived index, may have potential value for population-level risk stratification. While it is not intended as a diagnostic tool, incorporating such indices into routine health assessments may help inform targeted prevention strategies and support early intervention efforts in high-risk groups.

## Supplementary Information

Supplementary Material 1.

Supplementary Material 2.

## Data Availability

The datasets analyzed during the current study are publicly available from the China Health and Retirement Longitudinal Study (CHARLS) repository at https://charls.charlsdata.com/.
