# Supplementary material for: Baseline atherogenic coefficient and risk of incident chronic liver disease: a prospective cohort study from the CHARLS
Source: BMC Gastroenterol. 2026 Aug 6;26:725. doi: 10.1186/s12876-026-05176-8 (PMC13729408; doi:10.1186/s12876-026-05176-8)
Supplement: Supplementary file 1 [file 12876_2026_5176_MOESM2_ESM.docx]

**Supplementary Table S1. Baseline characteristics of study participants according to incident chronic liver disease status**

| **Characteristics** | **Total**  **Sample (n=7,452)** | **No**  **CLD (n=6,825)** | **Incident**  **CLD (n=627)** | **P value** |
| --- | --- | --- | --- | --- |
| **Continuous variables** |  |  |  |  |
| Age (years) | 58.28 ± 8.58 | 58.26 ± 8.62 | 58.50 ± 8.05 | 0.501 |
| BMI (kg/m²) | 23.65 ± 3.95 | 23.61 ± 3.92 | 24.08 ± 4.02 | 0.009 |
| Total cholesterol (mg/dL) | 194.13 ± 38.87 | 193.90 ± 38.28 | 196.68 ± 44.77 | 0.086 |
| HDL-C (mg/dL) | 51.15 ± 15.33 | 51.22 ± 15.22 | 50.31 ± 16.31 | 0.155 |
| Atherogenic coefficient | 3.12 ± 1.67 | 3.10 ± 1.53 | 3.38 ± 2.74 | <0.001 |
| **Categorical variables** |  |  |  |  |
| Age group, n (%) |  |  |  | 0.188 |
| 45–60 years | 4,709 (63.19) | 4,328 (63.41) | 381 (60.77) |  |
| >60 years | 2,743 (36.81) | 2,497 (36.59) | 246 (39.23) |  |
| ADL impairment, n (%) | 1,106 (15.01) | 972 (14.40) | 134 (21.65) | <0.001 |
| IADL impairment, n (%) | 1,373 (18.51) | 1,238 (18.23) | 135 (21.57) | 0.040 |
| Male sex, n (%) | 3,314 (44.50) | 3,012 (44.15) | 302 (48.24) | 0.049 |
| Education level, n (%) |  |  |  | 0.001 |
| Illiterate | 2,137 (28.70) | 2,001 (29.34) | 136 (21.76) |  |
| Primary school | 3,034 (40.75) | 2,755 (40.39) | 279 (44.64) |  |
| Middle school | 2,187 (29.37) | 1,986 (29.12) | 201 (32.16) |  |
| College or above | 88 (1.18) | 79 (1.16) | 9 (1.44) |  |
| Marital status, n (%) |  |  |  | 0.833 |
| Unmarried/divorced/widowed | 1,055 (14.16) | 968 (14.18) | 87 (13.88) |  |
| Married | 6,397 (85.84) | 5,857 (85.82) | 540 (86.12) |  |
| Current smoker, n (%) | 2,098 (28.59) | 1,935 (28.78) | 163 (26.55) | 0.242 |
| Current drinker, n (%) | 2,417 (32.45) | 2,210 (32.40) | 207 (33.01) | 0.751 |
| Household registration, n (%) |  |  |  | 0.001 |
| Rural | 6,321 (84.83) | 5,819 (85.27) | 502 (80.06) |  |
| Urban | 1,130 (15.17) | 1,005 (14.73) | 125 (19.94) |  |
| Depression, n (%) | 2,654 (37.02) | 2,389 (36.36) | 265 (44.31) | <0.001 |
| Poor self-rated health, n (%) | 5,864 (78.74) | 5,335 (78.23) | 529 (84.37) | <0.001 |
| Low life satisfaction, n (%) | 1,018 (15.53) | 906 (15.09) | 112 (20.33) | 0.001 |
| Chronic disease burden, n (%) |  |  |  | <0.001 |
| None | 2,385 (32.00) | 2,243 (32.86) | 142 (22.65) |  |
| One chronic disease | 2,233 (29.97) | 2,084 (30.53) | 149 (23.76) |  |
| Multiple chronic diseases | 2,834 (38.03) | 2,498 (36.60) | 336 (53.59) |  |
| Number of children, n (%) |  |  |  | 0.046 |
| No children | 1,889 (25.35) | 1,747 (25.60) | 142 (22.65) |  |
| One child | 1,819 (24.41) | 1,667 (24.42) | 152 (24.24) |  |
| Two children | 1,656 (22.22) | 1,490 (21.83) | 166 (26.48) |  |
| multiple children | 2,088 (28.02) | 1,921 (28.15) | 167 (26.63) |  |
| Atherogenic coefficient categories, n (%) |  |  |  | 0.004 |
| Low risk | 4,102 (55.05) | 3,774 (55.30) | 328 (52.31) |  |
| Mild risk | 1,755 (23.55) | 1,618 (23.71) | 137 (21.85) |  |
| Moderate risk | 920 (12.35) | 839 (12.29) | 81 (12.92) |  |
| High risk | 675 (9.06) | 594 (8.70) | 81 (12.92) |  |

Abbreviations: CLD, chronic liver disease; BMI, body mass index; HDL-C, high-density lipoprotein cholesterol; ADL, activities of daily living; IADL, instrumental activities of daily living.

**Supplementary Table S2. Cox regression models for the association between the atherogenic coefficient and Chronic Liver Disease**

| **Subgroup** | **Categories** | **Model 1**  **(Unadjusted)** |  |  | **Model 2**  **(Adjusted for age, gender)** |  |  | **Model 3**  **(Fully adjusted)** |  |  |
| --- | --- | --- | --- | --- | --- | --- | --- | --- | --- | --- |
|  | **Risk groups** | HR | 95% CI | *P* | HR | 95% CI | *P* | HR | 95% CI | *P* |
| **Overall sample** | **Normal group** | **1(Reference)** |  |  | **1(Reference)** |  |  | **1(Reference)** |  |  |
|  | **Mild risk group** | 0.97 | 0.80–1.19 | 0.787 | 0.98 | 0.81–1.20 | 0.868 | 1.00 | 0.81–1.24 | 0.998 |
|  | **Moderate risk group** | 1.10 | 0.87–1.40 | 0.417 | 1.11 | 0.88–1.42 | 0.379 | 1.03 | 0.78–1.35 | 0.850 |
|  | **High risk group** | 1.52 | 1.20–1.93 | 0.001 | 1.54 | 1.21–1.95 | 0.001 | 1.54 | 1.17–2.00 | 0.002 |

Model 1: unadjusted.

Model 2: adjusted for age and sex.

Model 3: adjusted for Model 2 plus BMI, smoking status, alcohol consumption, household registration type, educational level, activities of daily living, and depressive status.

**Model 3’:** adjusted for Model 3 without alcohol consumption.

Atherogenic coefficient: the ratio obtained by dividing the difference between total cholesterol and high-density lipoprotein cholesterol by high-density lipoprotein cholesterol.; HR: hazard ratio; CI: confidence interval; BMI: body mass index

**Supplementary Table S3. Interval-censored Cox proportional hazards models for the association between atherogenic coefficient and incident chronic liver disease**

| **Subgroup** | **Categories** | **Model 1**  **(Unadjusted)** |  |  | **Model 2**  **(Adjusted for age, gender)** |  |  | **Model 3**  **(Fully adjusted)** |  |  |
| --- | --- | --- | --- | --- | --- | --- | --- | --- | --- | --- |
|  | **Risk groups** | HR | 95% CI | *P* | HR | 95% CI | *P* | HR | 95% CI | *P* |
| **Overall sample** | **Normal group** | **1(Reference)** |  |  | **1(Reference)** |  |  | **1(Reference)** |  |  |
|  | **Mild risk group** | 0.97 | 0.80–1.19 | 0.785 | 0.98 | 0.80–1.20 | 0.865 | 1.00 | 0.80–1.25 | 0.992 |
|  | **Moderate risk group** | 1.10 | 0.87–1.41 | 0.416 | 1.12 | 0.87–1.42 | 0.380 | 1.02 | 0.78–1.36 | 0.851 |
|  | **High risk group** | 1.53 | 1.20–1.95 | 0.001 | 1.55 | 1.21–1.97 | 0.001 | 1.55 | 1.18–2.03 | 0.002 |

Model 1: unadjusted.

Model 2: adjusted for age and sex.

Model 3: adjusted for Model 2 plus BMI, smoking status, alcohol consumption, household registration type, educational level, activities of daily living, and depressive status.

Atherogenic coefficient: the ratio obtained by dividing the difference between total cholesterol and high-density lipoprotein cholesterol by high-density lipoprotein cholesterol.; HR: hazard ratio; CI: confidence interval; BMI: body mass index
